# Development of Pressure-Responsive PolyPropylene and Biochar-Based Materials

**DOI:** 10.3390/mi11040339

**Published:** 2020-03-25

**Authors:** Amir Noori, Mattia Bartoli, Alberto Frache, Erik Piatti, Mauro Giorcelli, Alberto Tagliaferro

**Affiliations:** 1 Department of Applied Science and Technology, Polytechnic of Turin, Alessandria Branch, Viale Teresa Michel 5, 15121 Alessandria, Italy; amir.noori@polito.it; 2 Department of Applied Science and Technology, Polytechnic of Turin, C.so Duca degli Abruzzi 24, 10129 Torino, Italy; mattia.bartoli@polito.it (M.B.); erik.piatti@polito.it (E.P.); mauro.giorcelli@polito.it (M.G.); 3 Faculty of Science, University of Ontario Institute of Technology, Oshawa, ON L1G 0C5, Canada

**Keywords:** biochar, PP, conductivity, composites, sensor

## Abstract

In this research paper, we reported the synthesis of biochar-based composites using biochar derived from exhausted tea leaves and polypropylene. The resulting materials were deeply characterized investigating mechanical (dynamic mechanical thermal analysis), thermal (thermogravimetrical analysis and differential scanning calorimetry), morphological (field emission scanning microscopy) and electrical properties vs. temperature. Furthermore, electrical conductivity was studied for a wide range of pressures showing an irreversible plastic deformation. An increment of one order of magnitude in the conductivity was observed in the case of 40 wt% biochar loading, reaching a value of 0.2 S/m. The material produced exhibited the properties of an irreversible pressure sensor.

## 1. Introduction

Tea production is one of the largest beverage commodities with an annual production of up to 4.6 Mton/y reached in 2010 [1]. During the preparation of tea beverage, the extraction process removes only a very small amount of compounds leaving a massive wet residue [2,3,4]. This waste stream could represent a challenge and an opportunity at the same time. Many applications have been found for exhausted tea leaves, ranging from composting [5,6,7] to adsorptive processes [8,9,10,11]. Other researchers have proposed the recovery of bioactive chemicals through additional extraction from exhausted tea residues [12,13,14]. Furthermore, Uzun et al. [15] reported the pyrolytic conversion of tea for the simultaneous production of bio-oils and biochar. Bio-oils are poor biofuel mixtures due to the massive presence of water [16] and a very complex chemicals platform. Conversely, biochar represents a very attractive carbon source for material science [17]. Biochar has been used in many applications but one of the most attractive has been the production of composites [18]. Reinforced plastic materials have been widely used in several key sectors of industry such as aerospace [19] and automotive [20]. In the field of industry, thermoplastic-based composites are largely used with an annual production of up to 29.5% of the overall production [21]. Several studies have been reported about the production of biochar containing polyethylene [22,23,24] or polypropylene [25,26,27,28] that enhanced the mechanical and thermal properties of the host polymeric matrix. Nonetheless, few research efforts have been devoted to investigate the effect of biochar produced at high temperatures towards the enhancement of polymer conductivity [29]. This field has conducted in-depth studies on using thermoset-based composites [30,31,32,33] or carbon nanotubes-based thermoplastic polymeric hosts [34,35,36,37,38] but it has neglected to consider thermoplastic and biochar at the same time. The use of a thermoplastic polymer matrix could be very useful for the production of irreversible resistive sensors that could detect the plastic deformation of the material at lower pressure ranges than thermoset-based sensors. These materials are very useful in the production of safety systems for both pedestrian [39] and vehicle [40] transportation. Similar results could be achieved using high-tech and very expensive carbon-based fillers (e.g., carbon nanotubes [41] and graphene [42]) or high loading of high-quality carbon black [43,44].

In this study, we developed a tea-derived biochar for the production of polypropylene (PP) composites with a filler loading of 30 wt% and 40 wt%. First, we first studied the thermal, mechanical, and electrical properties of both biochar and related composites. Afterwards, we compared them with carbon black containing PP with a filler loading of up to 40 wt%. Our aim was to demonstrate the feasibility of using biochar for the realization of resistive sensors acting under irreversible plastic deformation. 

## 2. Materials and Methods 

### 2.1. Materials

PP with a melt flow index (MFI) of 12 g/10 min, 2.16 kg, 230 °C, and density of 0.90 g/cm³ under the commercial name of Moplen HP500N was supplied by LyondellBasell (London, UK).

Tea leaves were recovered after the preparation of the beverage. Prior the pyrolysis, they were dried at 105 °C for 72 h. Exhausted tea leaves (100 g) were pyrolyzed using a vertical furnace and a quartz reactor, heating rate of 15 °C/min, and kept at 1000 °C for 30 min in an argon atmosphere. 

Commercial carbon black (VULCAN^®^ 9 N115, Cabot, Port Dickson, Malaysia) was used as a reference to be compared with exhausted tea leaves biochar.

### 2.2. Methods

All samples were investigated from the morphological point of view using field emission scanning electrical microscopy (FESEM, Zeis SupraTM 40, Oberkochen, Germany). The microscope was equipped with an energy dispersive X-Ray detector (EDX, Oxford Inca Energy 450, Oberkochen, Germany) that was used to explore the composition of the biochar.

PP and two carbon-based fillers, namely biochar derived from tea and carbon black, with different filler content were melt blended by means of a corotating twin screw micro extruder (DSM Xplore, 15 mL Microcompounder model, Arendstraat 5, Sittard, The Netherlands). The micro extruder consisted of a divisible fluid-tight mixing compartment and two detachable, conical mixing screws. Residence time could be modified via recirculation of the melt and remained constant, for all the runs, at 3 min. In order to avoid the degradation of the polymer during the processing time, a N_2_ purge flow was used. The screw speed was fixed at 50 rpm for feeding, and 100 rpm for the melt mixing, and the heating temperature was set at 190 °C. 

The specimens for dynamic mechanical thermal analyses (DMTA) were produced using a hot compression molding press at a heating temperature of 190 °C and 80 bar of pressure, for 3 min, obtaining 60 × 60 × 1 mm^3^ plates. The final specimens for tests (6 × 30 × 1 mm^3^) were derived from plates by razor blade cutting.

The thermogravimetric analysis (TGA) was conducted using a Discovery TA Instruments (New Castle, DE, USA) analyzer. The samples (ca. 10 mg) were placed in open alumina pans, heated from 50 to 700 °C at 10 °C/min rate, with a nitrogen flow of 25 mL/min. The data collected were *T_onset5%_* (temperature of 5% weight loss), *T_max_* (temperature of maximum weight loss rate), amount of the residue at 700 °C. 

The differential scanning calorimetry (DSC) analyses were carried out with a DSC Q20 supplied by TA Instruments (New Castle, DE, USA). First, samples of about 8 mg were heated at 10 °C/min under nitrogen from −50 to 220 °C in order to erase the previous thermal history. Then, after a 3 min isothermal step at 220 °C, samples were cooled at 10 °C/min to −50 °C, and finally reheated to 220 °C at 10 °C/min. The percentage crystallinity (*X_c_*) of neat polymers and composites was calculated using the following equation:

$$\chi_{c}\left(\%\right)=\frac{\Delta H_{m}}{\Delta H_{100}\left(1-x\right)}*100$$

where *ΔH_m_* is the melt enthalpy obtained from the second heating cycle as the integral of the area under heat flow curve, *ΔH_100_* represents the melting enthalpy of the 100% crystalline polymer (207 J/g for PP), and *x* is the filler weight percentage.

Dynamic mechanical thermal analysis (DMTA) was conducted using a DMA Q800 TA Instruments (New Castle, DE, USA). The following experimental conditions were selected: temperature range from room temperature to 150 °C in air, heating rate of 3 °C/min, 1 Hz frequency, and 0.05% of oscillation amplitude in strain-controlled mode. The storage modulus (E’) was measured with a tension film clamp on 6 × 30 × 1 mm^3^ samples.

Electric transport measurements on biochar derived from exhausted tea leaves were performed in the four-wire configuration by electrically contacting the biochar samples with thin gold wires and conducting silver paste. A constant current, *I* = 100 μA, was applied between the outer current contacts with a B2912 source-measure unit, and the longitudinal voltage drop that occurred across the inner voltage contacts, *V*_xx_, was measured with a 34420 nanovoltmeter (Keysight Technologies, Santa Rosa, CA, USA). Thermoelectric voltages were removed by inverting the sourced current within each resistance measurement. The temperature dependence of the electric resistance, *R* = *V*_xx_/*I*, was measured by loading the samples in the high-vacuum chamber of an ST-403 pulse-tube cryocooler (Cryomech, Syracuse, NY, USA), cooling the system to the base temperature of 2.7 K, and then allowing the samples to quasi-statically heat up to 300 K due to the residual thermal coupling to the outside environment. Then, the sheet resistance was calculated as *R*_s_ = *R* × *w*/*l*, where *w* and *l* are the length and width of the sample between the voltage contacts, respectively, and the sheet conductance as *G*_s_ = 1/*R*_s_.

The electrical conductivity of powder and composites was measured according to Gabhi et al. [45]. The equipment was composed of two solid copper cylinders (30 mm in diameter and 5 cm in length) encapsulated in a hollow homemade Plexiglas cylinder (inner diameter of 30 mm) in the case of filler electrical characterization. In this configuration, the inner diameter was slightly higher, and therefore it was possible to force the copper rods inside the Plexiglas cavity and the upper rod could slide inside the cylinder during the measurement. This arrangement created an internal chamber between the two cylinders, where the carbon powder was trapped. In case of composites, the Plexiglas cylinder was removed, and the sample was positioned between the aligned copper cylinders. The specimens for tests (6 × 30 × 3 mm^3^) were derived from plates by razor blade cutting.

The electrical resistance of powders and composites was measured under increasing loads (up to 1500 bar) applied by a hydraulic press (Specac Atlas Manual Hydraulic Press 15T, Orpington, UK). Electrically insulating sheets were placed between the conductive cylinders and the load surfaces in order to ensure that the electrical signal went through the sample. The resistance of the carbon fillers was measured using an Agilent 34401A multimeter (Keysight Technologies, Santa Rosa, CA, USA). 

## 3. Results

### 3.1. Characterization of Feedstock and Biochar

**Figure 1:**
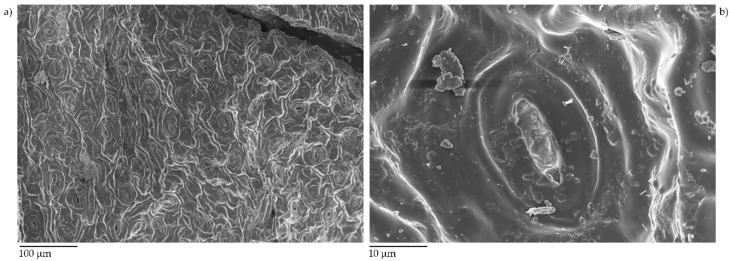
Capture of untreated exhausted tea leaves after metalization with 5 nm layer of chromium with low (**a**) and high (**b**) magnification.

Firstly, we investigated the compositional and morphological structure of exhausted tea leaves through FESEM techniques, as shown in Figure 1.

**Figure 2:**
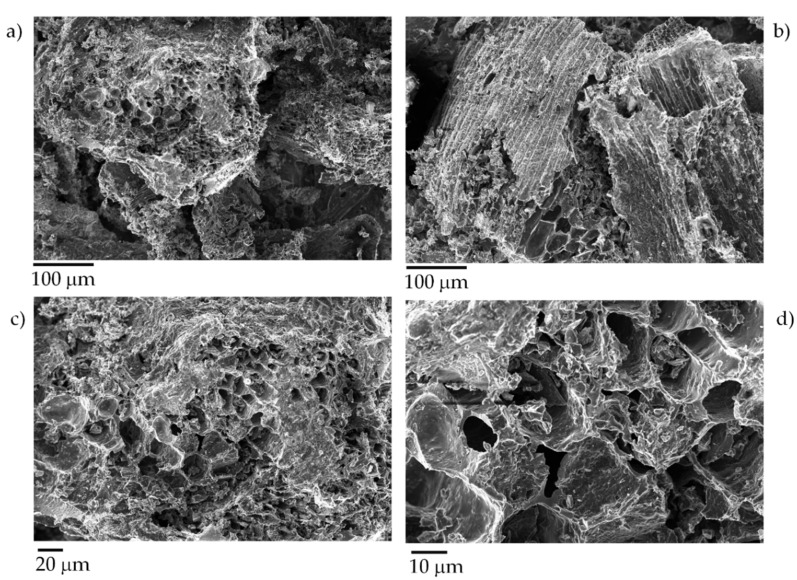
Captures using different magnification (**a**,**b**) 0.5 k, (**c**) 1.5 k, (**d**) 3 k of tea biochar produced at 1000 °C.

Exhausted tea leaves showed a typical leaves structure with stomata with an outer diameter up to 20 µm and an inner one around 10 µm. After the pyrolytic process, exhausted tea leaves underwent a drastic modification, as shown in Figure 2.

Biochar produced from tea leaves showed a channeled surface with thin inter-channels walls up to 1 µm. The pore diameter ranged from 10 to 20 µm. The channel structure was detected only in pyrolyzed leaves due the expansion of the channels, as reported by Bartoli et al. [46]. In this case, the pores’ collapse was balanced by the massive release of volatile organic matter from the inner core of the materials avoiding a reduction of pore diameter.

Another appreciable modification between unpyrolyzed leaves (exhausted tea leaves) and biochar is represented by the chemical composition, studied by EDX, as shown in Table 1. 

Exhausted tea leaves showed a low content of carbon and a high amount of oxygen together with a relevant amount of alkaline and alkaline earth metals [47]. After pyrolysis, biochar showed a significant increase of carbon content, up to 73.3 wt%, together with a decrement of oxygen to 16.4 wt%. After degradation of the original organic structure, the inorganic amount was magnified and traces of aluminum, sulphur, and phosphorous were detected. Trace amounts of these elements were present in the original but they were beyond the detection limit prior the pyrolysis, and they became appreciable only after carbonization. 

### 3.2. Characterization of Biochar-Based Composites

#### 3.2.1. Mechanical and Thermal Properties

**Figure 3:**
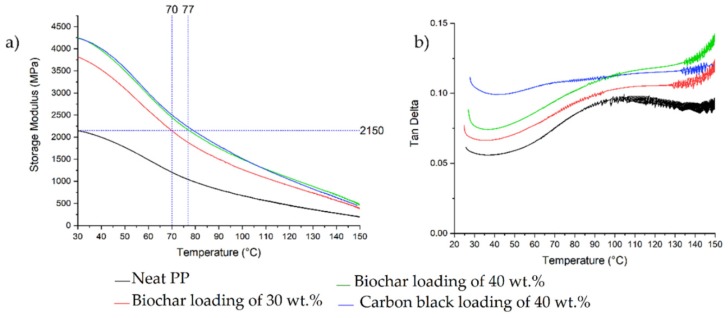
Storage modulus (**a**) and tan delta (**b**) of different composites containing biochar with a loading of 30 wt%, 40 wt%, and carbon black with a loading of 40 wt%.

DMTA is a technique that quantifies the properties of materials as they are deformed under periodic stress and its results are generally represented in terms of the complex modulus, which is defined as follows:

$$E^{*}=E^{\prime }+iE^{\prime \prime }$$

where E* is the complex modulus, *E′* is the storage modulus, and *E″* the loss modulus. On the one hand, the storage modulus shows the stiffness of a viscoelastic material and is proportional to the stored energy during a loading cycle; on the other hand, loss modulus is defined to be proportional to the dissipated energy [48]. The ratio between *E″* and *E′* is called tan delta. The peak in tan delta curves is the ratio of the dissipated energy to the energy stored per cycle of sample deformation at the glass transition temperature. In Figure 3a, the variation of the storage modulus versus temperature for three composites with respect to neat PP is reported. 

At first glance, it can be clearly observed that storage modulus of PP containing carbon black or biochar at 40 wt% are almost identical, and therefore in terms of mechanical properties there is no difference in substitution of pyrolyzed tea for carbon black. The modulus of all the samples decreased with increasing temperature, which was caused by the increase in segmental PP chain motion with temperature. After the addition of 40 wt% of both fillers, the storage modulus at 30 °C almost doubled. Interestingly, at approximately 77 °C, the storage modulus of neat PP dropped to 1080 MPa; whereas, the composites with 40 wt% of both fillers showed a storage modulus of approximately 2150 MPa, close to that of pure PP at 30 °C. The loss factor reported, and shown in Figure 3b, showed the increment of dissipated energy to the energy stored per cycle of sample deformation with the increased of filler loading.

**Figure 4:**
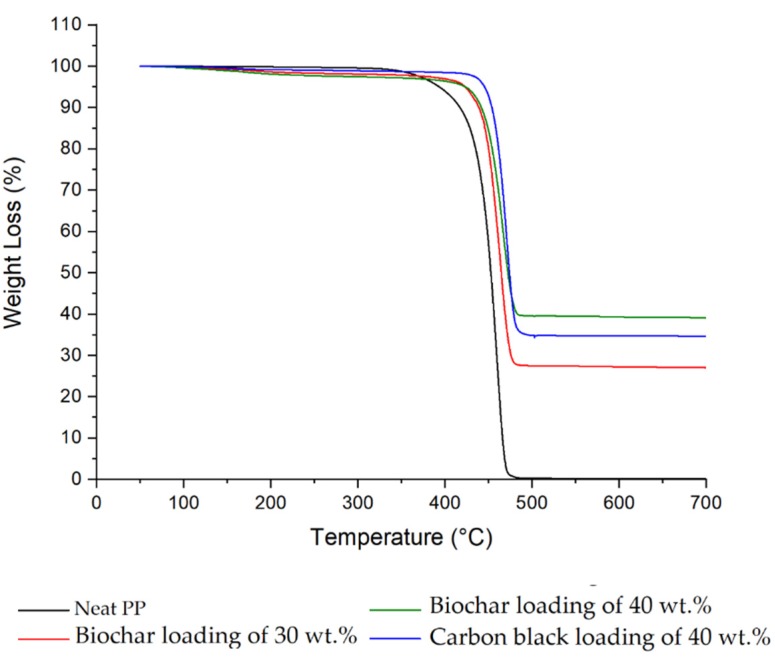
Thermal stability of different composites: neat polypropylene (PP), biochar (30 and 40 wt%), and carbon black (40 wt%).

TGAs were run in order to evaluate the thermal stability of the produced composites, as shown in Figure 4. The main outputs are summarized in Table 2.

It can be observed that the beginning of thermal degradation (*T*_onset5%_) was delayed 30 °C and 50 °C in both biochar composites and carbon black composite, respectively.

The temperature of maximum thermal degradation (*T*_max_) in pure PP is at 460 °C, whereas in the presence of both fillers, it is slightly shifted to higher temperatures. The residues at 700 °C show that the filler content is in accordance with what was expected.

All composites were tested through DSC analysis, as summarized in Table 3.

As expected, both fillers act as heterogeneous nucleants for PP and the temperature of crystallization has shifted 11 °C, from 117 °C in pure PP to approximately 128 °C in all composites. In terms of crystallinity, there is a slight increase in all composites with respect to pure PP.

#### 3.2.2. Electrical Properties 

**Figure 5:**
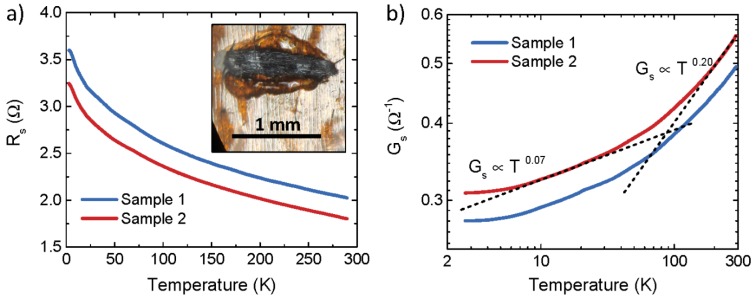
(**a**) Sheet resistance as a function of temperature for two different samples of biochar derived from exhausted tea leaves in linear scale. Inset is an optical image of biochar Sample 1 connected with gold wires and silver paste; (**b**) Corresponding sheet conductance as a function of temperature in log-log scale. Dashed lines indicate two different power-law scalings of the sheet conductance with temperature.

Firstly, two samples of the as-produced biochar were analyzed to determine the temperature dependence of their electrical sheet resistance, *R*_s_. Results are shown in Figure 5a.

*R*_s_ was found to weakly increase upon decreasing temperature, *T*, and showed an incipient saturation below 10 K, a behavior typical of disordered metallic systems [49,50,51]. This was quantitatively assessed by considering the scaling of *G*_s_ with *T* in log-log scale, as shown in Figure 5b. In addition to the incipient saturation at low *T*, the biochar samples showed two different power-law scalings ($G_{s}\propto T^{\beta }$) at intermediate (β≃0.07) and high temperatures (β≃0.2), with a crossover around T∼90 K. According to the theory of the insulator-to-metal transition (IMT), power-law dependences of the conductivity with temperature are typical of the quantum critical regime of the IMT, with β<1/3  and β>1/3 being associated with its metallic and insulating sides, respectively [49,52,53]. In the case of the biochar considered here, β<1/3 at any *T*, indicated metallic behavior. The presence of a crossover between two different power laws also suggests either the presence of two different conductive channels with a different metallicity, or a strong change in the temperature-dependence of the main source of charge-carrier scattering.

**Figure 6:**
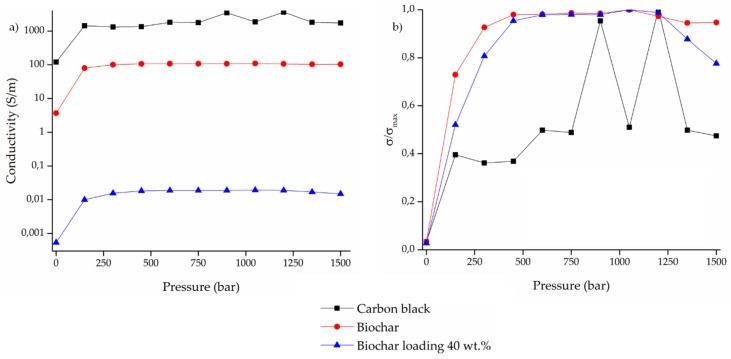
(**a**) Electrical conductivity measurement as a function of pressure on fillers and related composites containing biochar with a loading of 40 wt%, carbon black and tea biochar; (**b**) Electrical conductivity normalized on the highest value measured across the pressure range investigated.

Afterwards, both fillers and related epoxy composites were tested in a wide pressure range to evaluate the electrical conductivity, as shown in Figure 6.

First, we evaluated the conductivity of the fillers. The used methodology tightly compacted the powders and a trustworthy value was obtained. We evaluated the conductivity of the powders showing the better performance of carbon black upon biochar. Carbon black powder reached a conductivity of up to 1700 S/m, whereas, in the same conditions, biochar reached a conductivity of 105 S/m. This trend was not observed in the case of the composites. Composites filled with 40 wt% carbon black or with a biochar loading of up to 30 wt% were not conductive, whereas those produced using biochar loading showed a low conductivity of up to 5 × 10^−4^ S/m. The rapid conductivity increment between 1 and 500 bar is highlighted in Figure 6b, where the conductivity values were normalized. The conductivity of biochar and related composites increased faster than that of carbon black. Carbon black showed an interesting behavior at high pressure, with some fluctuation reasonably due to the packing process of nanometric carbon particles. The axial deformation reached 44% of the initial thickness of the 40 wt% biochar loaded samples under a pressure of 1500 bar, while the non-axial deformation was not appreciable. After the pressure was removed, the deformation did not disappear, and the thickness of the samples remained fixed at its high-pressure value. 

**Figure 7:**
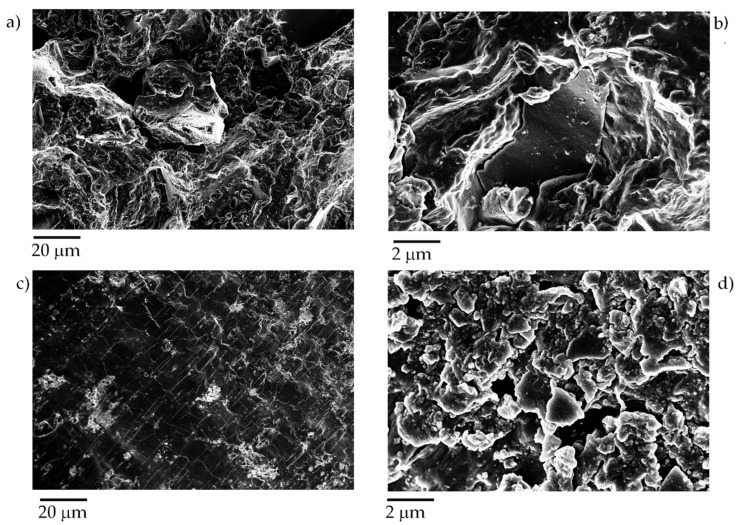
Captures using different magnification of composites containing (**a**,**b**) tea biochar produced at 1000 °C and (**c**,**d**) carbon black with a filler loading 40 wt%.

Furthermore, only the composite containing a biochar amount of up to 40 wt% showed an increment of conductivity of up to 0.02 S/m increasing the pressure. This behavior could be reasonably due to the dispersion of the PP into the polymeric matrix, as shown in Figure 7. 

Particles of biochar were well dispersed and reduced in size, similar to results previously reported using ultrasonication [54] instead of two-screws extrusion. Carbon black containing composites showed the presence of submicrometric aggregates well embedded into the PP matrix (Figure 7c,d). The extrusion led to the formation of isolated agglomerates of carbon black of approximately 10 µm in size, whereas the biggest detected particle of biochar had a size lower than 5 µm.

The good dispersibility of biochar is key to reaching the highest conductibility detected in the related composites and it is led by two main factors. The first factor is the lower π–π interactions occurring between biochar particles, rather than the interactions between carbon black particles. The second factor and advantage of biochar is due to its great fragmentation during the extrusion process that avoided the formation of conglomerate, as in the case of carbon black.

Biochar-based material showed a conductivity change together with an irreversible plastic deformation and could be employed in a large number of applications, as proven by a recent patent in the same field [55]. This behavior is very useful as a sensor for those applications where a fragile material cannot be used due to the sudden break event. In this case, PP-based composites could be a very interesting choice due to the combination of a greater plastic phase than common epoxy resins and better mechanical performances than silicon-based materials [56]. The combination of PP properties with a waste-derived filler, such as exhausted tea-derived biochar, is a very solid approach to be explored in the realm of pressure sensors. 

## 4. Conclusions

In this research work, we evaluated the use of exhausted tea leaves for the production of a conductive biochar. As-produced biochar samples were found to be weakly metallic down to low temperatures. We produced a powder with a remarkable conductivity of 105 S/m and we efficiently dispersed it into a PP matrix up to a loading of 40 wt%. The material showed a general improvement of mechanical and thermal properties. This was due to the amount of filler instead of the type of filler, because similar concentrations of carbon black and biochar induced similar effects.

With respect to the conductivity, the easier dispersibility of biochar is an obvious advantage over carbon black, which allows it to reach a conductivity up to 0.02 S/m. This phenomenon occurs together with a plastic deformation that freezes the composite structure, effectively acting as an irreversible pressure sensor. This technology should readily find an application, in many sectors, as a sensor to detect the local failure due to an impact. 

## Figures and Tables

**Table 1:** Elemental composition of exhausted tea leaves and related biochar.

Element	Composition (wt%)	
	Exhausted Tea Leaves	Biochar
C	31.8	73.3
O	58.2	16.4
Mg	2.6	0.5
Al	Not detected	0.5
Si	Not detected	0.2
P	Not detected	0.6
S	Not detected	0.3
Cl	Not detected	0.4
K	5.7	6.1
Ca	1.3	1.9

**Table 2:** The thermogravimetric analysis (TGA) data summary of biochar and carbon black containing composites.

Filler Loading (wt%)	Filler Type	*T*_onset 5%_ (°C)	*T*_max_ (°C)	Residue at 700 °C (%)
0	-	393	460	0
30	Biochar	423	463	27
40	Biochar	423	467	39
40	Carbon black	445	471	35

**Table 3:** Differential scanning calorimetry (DSC) data summary of biochar and carbon black containing composites.

Filler Loading (wt%)	Filler Type	*T*_C_ (°C)	$\Delta H_{c}\;(J/g)$	*T*_m_ (°C)	$\Delta H_{m}\;(J/g)$	**χ (%)**
0	-	117	109	165	110	53
30	Biochar	128	82	165	83	57
40	Biochar	129	65	165	66	53
40	Carbon Black	127	73	167	74	59

## References

[1] Basu Majumder A., Bera B., Rajan A. (2010). Tea statistics: Global scenario. Int. J. Tea Sci..

[2] Sanderson G.W. (1972). The Chemistry of Tea and Tea Manufacturing. Recent Advances in Phytochemistry.

[3] Robinson J., Owuor P.O. (1992). Tea Aroma. Tea.

[4] Yamanishi T., Kobayashi A. (1999). Progress of tea aroma chemistry. Flavor Chemistry.

[5] Khan M.A.I., Ueno K., Horimoto S., Komai F., Tanaka K., Ono Y. (2009). Physicochemical, including spectroscopic, and biological analyses during composting of green tea waste and rice bran. Biol. Fertil. Soils.

[6] Bess V.H. (2000). Understanding compost tea. BioCycle.

[7] Gao P., Ogata Y. Biodegradability of PLA and Tea Waste Composites Based on “CHAMU” and the “Tea Waste Recycling System”. Proceedings of IOP Conference Series: Materials Science and Engineering.

[8] Cay S., Uyanık A., Özaşık A. (2004). Single and binary component adsorption of copper (II) and cadmium (II) from aqueous solutions using tea-industry waste. Sep. Purif. Technol..

[9] Mondal M. (2009). Removal of Pb (II) ions from aqueous solution using activated tea waste: Adsorption on a fixed-bed column. J. Environ. Manag..

[10] Madrakian T., Afkhami A., Ahmadi M. (2012). Adsorption and kinetic studies of seven different organic dyes onto magnetite nanoparticles loaded tea waste and removal of them from wastewater samples. Spectrochim. Acta Part A Mol. Biomol. Spectrosc..

[11] Huang C., Zhang H., Zeng W., Ma J., Zhao S., Jiang Y., Huang C., Mao H., Liao Y. (2020). Enhanced fluoride adsorption of aluminum humate and its resistance on fluoride accumulation in tea leaves. Environ. Technol..

[12] Sui W., Xiao Y., Liu R., Wu T., Zhang M. (2019). Steam explosion modification on tea waste to enhance bioactive compounds’ extractability and antioxidant capacity of extracts. J. Food Eng..

[13] Senol A., Aydin A. (2006). Solid–liquid extraction of caffeine from tea waste using battery type extractor: Process optimization. J. Food Eng..

[14] Shalmashi A., Abedi M., Golmohammad F., Eikani M.H. (2010). Isolation of caffeine from tea waste using subcritical water extraction. J. Food Process Eng..

[15] Uzun B.B., Apaydin-Varol E., Ateş F., Özbay N., Pütün A.E. (2010). Synthetic fuel production from tea waste: Characterisation of bio-oil and bio-char. Fuel.

[16] Garcia-Perez M., Chaala A., Pakdel H., Kretschmer D., Roy C. (2007). Characterization of bio-oils in chemical families. Biomass Bioenergy.

[17] Bartoli M., Giorcelli M., Jagdale P., Rovere M., Tagliaferro A. (2020). A review of non-soil biochar applications. Materials.

[18] Aup-Ngoen K., Noipitak M. (2020). Effect of carbon-rich biochar on mechanical properties of PLA-biochar composites. Sustain. Chem. Pharm..

[19] Arif M., Asif M., Ahmed I. (2017). Advanced composite material for aerospace application—A review. Int. J. Eng. Mfg. Sci.

[20] Jacob A. (2014). Carbon fibre and cars–2013 in review. Reinf. Plast..

[21] Research G.V. Market Value of Composite Materials Worldwide from 2015 to 2024 (in Billion U.S. Dollars). https://www.statista.com/statistics/944471/global-market-value-of-composites/.

[22] Zhang Q., Yi W., Li Z., Wang L., Cai H. (2018). Mechanical properties of rice husk biochar reinforced high density polyethylene composites. Polymers.

[23] Arrigo R., Jagdale P., Bartoli M., Tagliaferro A., Malucelli G. (2019). Structure–property relationships in polyethylene-based composites filled with biochar derived from waste coffee grounds. Polymers.

[24] Zhang Q., Khan M.U., Lin X., Cai H., Lei H. (2019). Temperature varied biochar as a reinforcing filler for high-density polyethylene composites. Compos. Part B Eng..

[25] Das O., Bhattacharyya D., Hui D., Lau K.-T. (2016). Mechanical and flammability characterisations of biochar/polypropylene biocomposites. Compos. Part B Eng..

[26] Das O., Sarmah A.K., Bhattacharyya D. (2016). Biocomposites from waste derived biochars: Mechanical, thermal, chemical, and morphological properties. Waste Manag..

[27] Das O., Kim N.K., Hedenqvist M.S., Lin R.J., Sarmah A.K., Bhattacharyya D. (2018). An attempt to find a suitable biomass for biochar-based polypropylene biocomposites. Environ. Manag..

[28] Elnour A.Y., Alghyamah A.A., Shaikh H.M., Poulose A.M., Al-Zahrani S.M., Anis A., Al-Wabel M.I. (2019). Effect of pyrolysis temperature on biochar microstructural evolution, physicochemical characteristics, and its influence on biochar/polypropylene composites. Appl. Sci..

[29] Li S., Huang A., Chen Y.-J., Li D., Turng L.-S. (2018). Highly filled biochar/ultra-high molecular weight polyethylene/linear low density polyethylene composites for high-performance electromagnetic interference shielding. Compos. Part B Eng..

[30] Giorcelli M., Savi P., Khan A., Tagliaferro A. (2019). Analysis of biochar with different pyrolysis temperatures used as filler in epoxy resin composites. Biomass Bioenergy.

[31] Khan A., Savi P., Quaranta S., Rovere M., Giorcelli M., Tagliaferro A., Rosso C., Jia C. (2017). Low-cost carbon fillers to improve mechanical properties and conductivity of epoxy composites. Polymers.

[32] Savi P., Yasir M., Bartoli M., Giorcelli M., Longo M. (2020). Electrical and microwave characterization of thermal annealed sewage sludge derived biochar composites. Appl. Sci..

[33] Giorcelli M., Bartoli M. (2019). Development of coffee biochar filler for the production of electrical conductive reinforced plastic. Polymers.

[34] Tzounis L., Hegde M., Liebscher M., Dingemans T., Pötschke P., Paipetis A.S., Zafeiropoulos N.E., Stamm M. (2018). All-aromatic SWCNT-Polyetherimide nanocomposites for thermal energy harvesting applications. Compos. Sci. Technol..

[35] Liebscher M., Gärtner T., Tzounis L., Mičušík M., Pötschke P., Stamm M., Heinrich G., Voit B. (2014). Influence of the MWCNT surface functionalization on the thermoelectric properties of melt-mixed polycarbonate composites. Compos. Sci. Technol..

[36] Yee M.J., Mubarak N., Khalid M., Abdullah E., Jagadish P. (2018). Synthesis of polyvinyl alcohol (PVA) infiltrated MWCNTs buckypaper for strain sensing application. Sci. Rep..

[37] Funck A., Kaminsky W. (2007). Polypropylene carbon nanotube composites by in situ polymerization. Compos. Sci. Technol..

[38] Tzounis L., Gärtner T., Liebscher M., Pötschke P., Stamm M., Voit B., Heinrich G. (2014). Influence of a cyclic butylene terephthalate oligomer on the processability and thermoelectric properties of polycarbonate/MWCNT nanocomposites. Polymer.

[39] Scherf O. Development and Performance of Contact Sensors for Active Pedestrian Protection Systems. Proceedings of the International Technical Conference on the Enhanced Safety of Vehicles.

[40] Schoeneburg R., Breitling T. Enhancement of Active and Passive Safety by Future PRE-SAFE^®^ Systems. Proceedings of the International Technical Conference on the Enhanced Safety of Vehicles.

[41] Valentino O., Sarno M., Rainone N.G., Nobile M.R., Ciambelli P., Neitzert H.C., Simon G.P. (2008). Influence of the polymer structure and nanotube concentration on the conductivity and rheological properties of polyethylene/CNT composites. Phys. E Low Dimens. Syst. Nanostruct..

[42] Fim F.D.C., Basso N.R., Graebin A.P., Azambuja D.S., Galland G.B. (2013). Thermal, electrical, and mechanical properties of polyethylene–graphene nanocomposites obtained by in situ polymerization. J. Appl. Polym. Sci..

[43] Cao Q., Song Y., Tan Y., Zheng Q. (2009). Thermal-induced percolation in high-density polyethylene/carbon black composites. Polymer.

[44] Petrović Z.S., Martinović B., Divjaković V., Budinski–Simendić J. (1993). Polypropylene–carbon black interaction in conductive composites. J. Appl. Polym. Sci..

[45] Gabhi R.S., Kirk D.W., Jia C.Q. (2017). Preliminary investigation of electrical conductivity of monolithic biochar. Carbon.

[46] Bartoli M., Nasir M.A., Jagdale P., Passaglia E., Spiniello R., Rosso C., Giorcelli M., Rovere M., Tagliaferro A. (2019). Influence of pyrolytic thermal history on olive pruning biochar and related epoxy composites mechanical properties. J. Compos. Mater..

[47] Milani R.F., Morgano M.A., Saron E.S., Silva F.F., Cadore S. (2015). Evaluation of direct analysis for trace elements in tea and herbal beverages by ICP-MS. J. Braz. Chem. Soc..

[48] Wetton R., Ruff P., Gearing J. (1987). Dynamic mechanical thermal analysis techniques in composites evaluation. Composites Evaluation.

[49] Heeger A.J. (2002). The critical regime of the metal-insulator transition in conducting polymers: Experimental studies. Phys. Scr..

[50] Mott N., Davis E. (1979). Electronic Processes in Non-Cyrstalline Materials.

[51] Eschrig H.N.F. (1991). Mott Metal-insulator transition. Taylor & Francis, London 1990, × + 286 pages, 166 figures, ISBN 0-85066-783-6. Cryst. Res. Technol..

[52] Piatti E., Galanti F., Pippione G., Pasquarelli A., Gonnelli R.S. (2019). Towards the insulator-to-metal transition at the surface of ion-gated nanocrystalline diamond films. Eur. Phys. J. Spec. Top..

[53] Piatti E., Romanin D., Daghero D., Gonnelli R.S. (2019). Two-dimensional hole transport in ion-gated diamond surfaces: A brief review. Low Temp. Phys..

[54] Bartoli M., Giorcelli M., Rosso C., Rovere M., Jagdale P., Tagliaferro A. (2019). Influence of commercial biochar fillers on brittleness/ductility of epoxy resin composites. Appl. Sci..

[55] Parker R. (2019). Printable High Pressure Irreversible Indicating Material. US Patent.

[56] Van Krevelen D., Te Nijenhuis K. (1990). Polymer properties. Prop. Polym..

